# The value of postoperative contrast-enhanced ultrasound parameters in lymph node metastasis, tumor node metastasis, and treatment response evaluation of resected hepatocellular carcinoma

**DOI:** 10.1097/MD.0000000000040108

**Published:** 2024-10-18

**Authors:** Chenhao Ying, Yefei Yao, Binjun Yang, Haijing Song

**Affiliations:** a Department of Ultrasound, The First People’s Hospital of Yongkang City, Yongkang, Zhejiang Province, China; b Department of Ultrasound, Hangzhou Hospital of Traditional Chinese Medicine, Hangzhou City, Zhejiang Province, China; c Department of Radiotherapy, The First People’s Hospital of Yongkang, Yongkang, Zhejiang Province, China.

**Keywords:** contrast-enhanced ultrasound parameters, hepatocellular carcinoma, lymph node metastasis, post-treatment response evaluation, tumor node metastasis

## Abstract

To explore the application value of postoperative contrast-enhanced ultrasound (CEUS) parameters for lymph node metastasis (LNM), tumor, node, metastasis staging, and treatment response evaluation of resected hepatocellular carcinoma (HCC). We retrospectively analyzed 100 patients with liver cancer who underwent liver CEUS at our hospital between October 2020 and October 2022. The patient’s LNM, pathological staging, and therapeutic effects were recorded based on the histopathological results. CEUS parameters were analyzed and compared CEUS parameters between different lymph node metastases, pathological stages, and therapeutic effects. Twenty-three patients experienced LNM, 77 patients did not experience LNM; and the rise time (RT), peak intensity (PI), and area under the curve (AUC) of the metastatic group were significantly smaller than those of the nonmetastatic group (*P* < .05). 44 cases were classified into groups I to II by pathological staging, and 56 cases were classified into groups III to IV. The RT, PI, and AUC of groups III to IV were significantly lower than those of groups I–II (*P* < .05). Seventy-nine cases were complete necrosis, 21 cases were residual or recurrent; The RT, PI, and AUC of the residual or recurrent group were significantly lower than those of the complete necrosis group (*P* < .05). The receiver operating characteristic curve shows that RT, PI, and AUC have a certain value in evaluating LNM, pathological staging, and treatment response of HCC, and the combined evaluation/evaluation value of these 3 factors is relatively high. The postoperative CEUS parameters RT, PI, and AUC can be used for LNM, pathological staging evaluation, and treatment response evaluation of HCC. Moreover, the combination of the 3 parameters is feasible and valuable in evaluating LNM, tumor, node, metastasis staging, and treatment response of HCC.

## 1. Introduction

Hepatocellular carcinoma (HCC) is a malignant tumor that occurs in liver cells or intrahepatic bile duct epithelial cells.^[1]^ HCC usually has no typical symptoms in the early stage, but when typical symptoms appear, most develop into the late stage.^[2]^ At present, the main treatment methods for HCC include surgical resection, vascular intervention, and radiofrequency ablation.^[1,2]^ However, regardless of the treatment method, the therapeutic effect is influenced by lymph node metastasis (LNM), tumor, node, metastasis (TNM) staging, and the patient’s physical condition.^[3]^ Therefore, early and accurate assessment of LNM and TNM staging in patients with HCC is particularly important for adopting appropriate treatment methods to improve clinical efficacy.^[1–3]^

As a commonly used screening method for malignant tumors, CEUS has the advantages of being noninvasive, economical, convenient, and reusable. It can clearly display the size, number, shape, boundary, echo status, and blood flow status of lesions.^[4–6]^ It has been widely used in LNM for thyroid cancer, breast cancer, and TNM staging assessments of cervical cancer.^[5–7]^ At present, many studies have reported the use of CEUS parameters in the diagnosis and treatment response evaluation of HCC.^[4,8]^ However, there are limited reports on the value of CEUS parameters in evaluating LNM and TNM staging in HCC. Postoperative relapse and metastasis of HCC are closely related to the therapeutic effect and patient survival. CEUS can dynamically observe the microvascular flow perfusion of the lesion in real time, and the postoperative application of CEUS can make accurate qualitative diagnosis of the recurrence and metastasis of the lesion, which is helpful for the early detection, diagnosis, and treatment of residual and recurrent tumors, so as to improve the therapeutic effect and patient survival period. Based on this, this study retrospectively analyzed the application value of CEUS parameters, such as rise time (RT), time to peak (TTP), peak intensity (PI), wash-in slope (WIS), area under the curve (AUC) in LNM, TNM evaluation, and treatment response evaluation of HCC to provide a theoretical basis for clinical diagnosis and treatment of HCC.

## 2. Materials and methods

We retrospectively selected 100 patients with HCC who underwent liver CEUS in our hospital between October 2020 and October 2022. Among them, 69 were males and 31 were females; The age range is 41 to 73 years, with a mean of 55.70 ± 6.40 years old; The diameter of the tumor is 3 to 5 cm, with a mean of 4.10 ± 0.54 cm; The body mass index ranges from 17.2 to 31.1 kg/m^2^, with a mean of 23.67 ± 2.75 kg/m^2^.

*Inclusion criteria:*

- Conforming to the diagnostic criteria of the “Guidelines for the Diagnosis and Treatment of Primary HCC (2022 Edition)” and confirmed by liver biopsy^[9]^
- No previous history of liver surgery;
- All the patients underwent surgical resection, lymph node dissection, and microwave/radiofrequency ablation.
- All have accepted CEUS and the image data has been saved completely;
- The medical record information is complete;
- Follow up records for at least 6 months.

*Exclusion criteria:*

- Patients with other malignant tumors;
- Patients with distant metastasis or secondary HCC;
- Pregnant and lactating patients.

*CEUS*: CEUS was performed 1 week, 1 month, 3 months, and 6 months postoperation. Using a GE LOGIQ E9 ultrasound diagnostic instrument, the probe frequency was 2.5 to 5.0 MHz, and the contrast agent was selected from Bracco, Italy’s sulfur hexafluoride microbubble because of its ability to enhance the image contrast of blood vessels. First, routine two-dimensional ultrasound examination was performed to primarily explore the patient’s liver portal vein system and its relationship with surrounding anatomical structures. Then, quickly inject 2.4 mL of the prepared sulfur hexafluoride microbubble suspension was quickly injected into the patient’s superficial vein at the elbow, and the tube was rinsed with 5 mL of physiological saline. Set the instrument to contrast-enhanced ultrasound mode and set the mechanical index to 0.08 to 0.12; Continuously scan the liver and intrahepatic biliary system from multiple angles, lasting for 5 to 6 minutes, to explore the perfusion of contrast agents in the liver parenchyma and portal vein ducts; If necessary, the target area can be repeatedly examined by contrast-enhanced ultrasound, and the interval between repeated examinations should not be <30 minutes. Store relevant images and use specialized image analysis software to obtain CEUS parameters such as RT, TTP, PI, WIS, and AUC. All CEUS and parameter measurements were performed by the same senior imaging physician.

Observation outcomes:

1. Lymph node metastasis conditions: Based on the presence or absence of LNM, patients were divided into 2 groups: patients without LNM and patients with LNM. The RT, TTP, PI, WIS and AUC of patients in both groups were compared.
2. Pathological stages: Based on the pathological stages of the tumor, patients were divided into stage I to II group and stage III to IV group. The RT, TTP, PI, WIS and AUC of patients in both groups were compared.
3. Treatment response evaluation: CEUS was used for follow-up examination 6 months posttreatment to evaluate treatment response. No enhancement was interpreted as complete tumor necrosis (complete response), that is, disappearance of the original lesion without the appearance of a new lesion. On the contrary, positive enhancement was interpreted as tumor residue/recurrence (incomplete response). The RT, TTP, PI, WIS and AUC of patients with tumor complete necrosis or tumor residue/recurrence were compared.
4. Application value of ultrasound parameters: Value of ultrasound parameters in LNM, pathological staging and treatment response of HCC were analyzed using the receiver operating characteristic (ROC) curve.

### 2.1. Statistical analysis

All data analyses were conducted using SPSS software (version 25.0; IBM Corp, Armonk, NY). The measurement data are expressed as mean ± standard deviation, and CEUS parameters were compared between patients with different lymph node metastases, between patients with different TNM stages, and between patients with different therapeutic effects using independent sample *t* tests. Use R4.2 for statistical analysis and plotting. Evaluate CEUS parameters RT, PI, and AUC using the ROC curve to predict LNM, TNM staging, and treatment response in patients. Statistical *P*-value less at *P* < .05, and all reported *p*-values were bilateral.

## 3. Results

**Figure 1.**
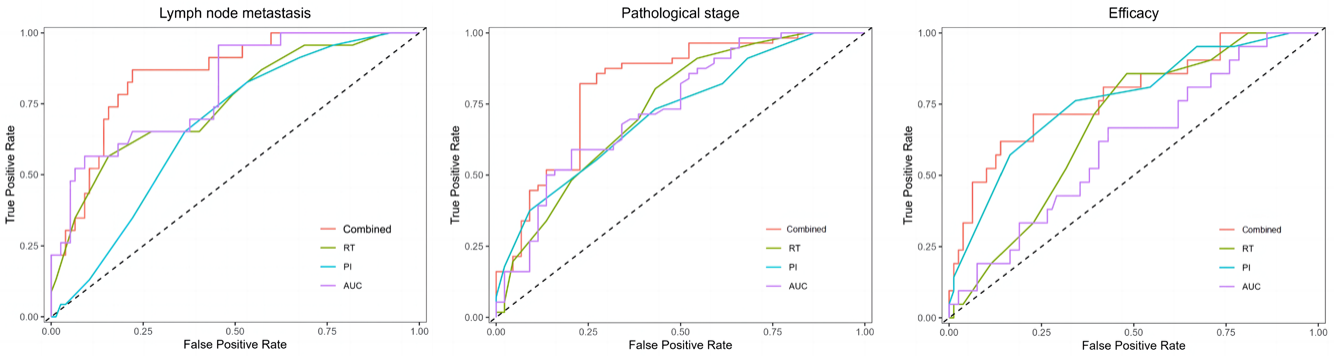
ROC curve of LNM, TNM staging, and treatment response in HCC patients evaluated by CEUS parameters such as RT, PI, AUC and their combination. AUC = area under the curve, CEUS = contrast-enhanced ultrasound, HCC = hepatocellular carcinoma, LNM = lymph node metastasis, PI = peak intensity, ROC = receiver operating characteristic RT = rise time, TNM = tumor, node, metastasis.

A total of 100 patients were included in this study, including 77 without LNM and 23 with LNM. The RT, PI, and AUC of LNM were significantly lower than those without LNM (*P* < .05); the comparison of TTP and WIS between the 2 was not statistically significant (*P* > .05) (Table 1). 44 cases were TNM classified as I to II, while 56 cases were TNM classified as III to IV. The RT, PI, and AUC of stage III to IV patients were significantly lower than those of stage I to II patients (*P* < .05), while the TTP and WIS of the 2 were not statistically significant (*P* > .05) (Table 2). After a follow-up of 6 months, 79 cases were complete necrosis and 21 cases were residual or recurrent; the residual or recurrent RT, PI, and AUC were significantly smaller than those in the complete necrosis group (*P* < .05), while there was no statistically significant difference in TTP and WIS between the 2 groups (*P* > .05) (Table 3). The ROC curve analysis results showed that the AUC of RT, PI, and AUC parameters for LNM, TNM staging evaluation, and treatment response evaluation were 0.752, 0.670, 0.802, 0.736, 0.719, 0.737, 0.679, 0.765, and 0.607, respectively; The maximum cutoff point of the Jordan index on the upper left side of the ROC curve is used as the optimal critical value. The sensitivities of this point for LNM, TNM staging evaluation, and treatment response evaluation were 56.5%, 82.6%, 87.0%, 80.4%, 73.2%, 58.9%, 71.4%, 76.2%, and 66.7, respectively. The specificity is 77.9%, 46.8%, 54.5%, 56.8%, 56.8%, 77.3%, 51.9%, 65.8%, and 57.0%, respectively, which has certain evaluation/evaluation value. Furthermore, ROC curve analysis showed that the AUC values for LNM, TNM staging evaluation, and treatment response evaluation were 0.852, 0.815, and 0.781, respectively, with sensitivities of 95.7%, 82.1%, and 85.7%, and specificities of 84.4%, 79.5%, and 77.2%, respectively, which were significantly higher than those for RT, PI, and AUC single detection. The combination of the 3 factors has a high value in evaluating LNM, TNM staging, and the therapeutic effect of HCC (Table 4 and Fig. 1).

**Table 1:** Comparison of ultrasound parameters in patients with/without LNM.

Group (n)	RT (s)	TTP (s)	PI (dB)	WIS	AUC
Patients without LNM (n = 77)	18.65 ± 2.85	35.92 ± 3.84	14.36 ± 2.35	1.10 ± 0.11	1628.65 ± 233.33
Patients with LNM (n = 23)	15.96 ± 2.80	36.09 ± 3.36	13.09 ± 1.73	1.08 ± 0.14	1338.57 ± 225.12
*t*	3.990	0.186	2.413	0.626	5.273
*P*	<.001	.853	.018	.533	<.001

AUC = area under the curve, LNM = lymph node metastasis, PI = peak intensity, RT = rise time, TTP = time to peak, WIS = wash-in slope.

**Table 2:** Comparison of ultrasound parameters in patients with different pathological stages.

Group (n)	RT (s)	TTP (s)	PI (dB)	WIS	AUC
I–II (n = 44)	19.43 ± 3.06	35.98 ± 3.78	15.07 ± 2.07	1.09 ± 0.12	1684.59 ± 255.77
III–IV (n = 56)	16.93 ± 2.56	35.95 ± 3.71	13.29 ± 2.14	1.10 ± 0.11	1465.55 ± 223.51
*t*	4.449	0.041	4.194	0.222	4.564
*P*	<.001	.967	<.001	.825	<.001

AUC = area under the curve, PI = peak intensity, RT = rise time, TTP = time to peak, WIS = wash-in slope.

**Table 3:** Comparison of ultrasound parameters in patients with different therapeutic effects.

Group (n)	RT (s)	TTP (s)	PI (dB)	WIS	AUC
Complete necrosis (n = 79)	18.43 ± 3.10	36.13 ± 3.73	14.52 ± 2.12	1.10 ± 0.12	1585.76 ± 257.81
Residual or recurrent (n = 21)	16.52 ± 2.32	35.33 ± 3.72	12.38 ± 2.11	1.09 ± 0.12	1472.29 ± 259.32
*t*	2.624	0.868	4.116	0.361	1.791
*P*	.010	.388	<.001	.719	.044

AUC = area under the curve, PI = peak intensity, RT = rise time, TTP = time to peak, WIS = wash-in slope.

**Table 4:** Value of ultrasound parameters in LNM, pathological staging and curative effect evaluation of HCC.

Ultrasonic parameters	AUC	S.E.	*P*	*95% C.I.*		Cutoff	Sensitivity	Specificity	Youden index
				Lower limit	Upper limit				
RT^①^	0.752	0.059	<.001	0.636	0.867	15.500	0.565	0.779	0.344
PI^①^	0.670	0.058	.014	0.557	0.783	14.500	0.826	0.468	0.294
AUC^①^	0.802	0.050	<.001	0.704	0.900	1588.500	0.870	0.545	0.415
Triplex	0.852	0.042	<.001	0.770	0.934	0.217	0.957	0.844	0.801
RT^②^	0.736	0.051	<.001	0.637	0.836	19.500	0.804	0.568	0.372
PI^②^	0.719	0.050	<.001	0.620	0.818	14.500	0.732	0.568	0.300
AUC^②^	0.737	0.050	<.001	0.638	0.836	1501.500	0.589	0.773	0.362
Triplex	0.815	0.044	<.001	0.728	0.903	0.535	0.821	0.795	0.616
RT^③^	0.679	0.058	.012	0.565	0.793	18.500	0.714	0.519	0.233
PI^③^	0.765	0.059	<.001	0.649	0.880	13.500	0.762	0.658	0.420
AUC^③^	0.607	0.067	.132	0.477	0.738	1540.500	0.667	0.570	0.237
Triplex	0.781	0.059	<.001	0.666	0.896	0.258	0.857	0.772	0.629

*Notes*: “①” is the parameter of LNM; “②” is the parameter of pathological stage; “③” is the parameter of curative effect.

AUC = area under the curve, LNM = lymph node metastasis, PI = peak intensity, RT = rise time.

## 4. Discussion

The results of this study indicate that CEUS parameters such as RT, PI, and AUC have value in evaluating LNM, TNM staging, and treatment response of HCC. However, when tested individually, the value is average, whereas when the 3 indicators are combined, their value for LNM, TNM staging evaluation, and treatment response evaluation is higher for HCC. CEUS was the first diagnostic method for solid tumors.^[4–8]^ HCC need to clarify their prognosis as soon as possible after treatment. Huang et al^[10]^ constructed a predictive model for the early recurrence of HCC after resection or ablation based on CEUS ultrasound features, and the results showed that the model had high sensitivity and specificity. The results of Cao et al^[11]^ also indicated that CEUS can be used to evaluate the early treatment response of advanced HCC patients to chemotherapy. This study analyzed the CEUS parameters of patients with HCC, and the results were consistent with the above research. In this study, 23 patients were diagnosed with LNM. LNM is the main mode of HCC metastasis and is an important reason for patients to choose surgical methods.^[12]^ Therefore, it is extremely important to determine whether patients with HCC have LNM prior to surgery. In normal tissues, lymph node blood supply is relatively scarce; however, once cancer cells undergo LNM, their blood supply becomes more abundant.^[12]^ Therefore, CEUS can be used to determine lymph node blood supply and diagnose LNM in cancer cells.^[12]^ Ultrasound has the advantages of being noninvasive, radiation-free, simple, and inexpensive, making it the preferred examination method for diagnosing LNM in patients with HCC.^[8–10]^ CEUS can dynamically observe angiogenesis and blood flow perfusion within a lesion by injecting a contrast agent into the peripheral blood and utilizing the strong backscatter signal generated by ultrasound contrast agent microbubbles in the blood. Yu et al^[13]^ found that static parameter imaging can describe dynamic blood flow perfusion patterns and serve as a guiding factor for improving the model’s classification ability. This finding is consistent with the results of the present study.

Accurate determination of TNM staging of HCC is extremely challenging for imaging.^[8,14]^ With the clinical application of new contrast agents, CEUS can accurately differentiate and determine TNM staging of HCC. The results of this study showed that the RT, PI, and AUC parameters of patients with HCC in stages III to IV were significantly lower than those of patients in stages I to II. CEUS can clearly display the distribution and blood flow grading of HCC lesion tissues and the surrounding blood vessels.^[4,14]^ The hepatic artery is the main blood supply artery for the HCC lesions. After injection of the contrast agent, it quickly appears as a mass and then exits. After liver parenchymal enhancement, the tumor shows weak echo, so fast in and out is the main enhancement feature. Cancer cells with a lower degree of deterioration are supplied by both the portal vein and hepatic artery, and contrast agents are continuously reinjected from the portal vein, which is characterized by fast in and slow out.^[15]^

Owing to the high sensitivity of CEUS for detecting blood flow signals within tumors. It has been used for many years for the differential diagnosis and evaluation of intralesional treatment of focal liver lesions. To identify residual areas of tumor enhancement or disease recurrence that may reflect incomplete necrosis.^[12]^ Therefore, CEUS parameters can be easily obtained and used to evaluate intrahepatic recurrence after radiofrequency ablation.^[14]^ The results of this study showed that the CEUS parameters such as RT, PI, and AUC in the residual or recurrent group were smaller than those in the complete necrosis group. The reason for this is that RT and TTP are indicators reflecting the blood supply composition of the lesion, which is determined by the blood supply composition of the lesion’s blood vessels, hepatic artery, and portal vein. The larger the proportion of the hepatic artery blood supply, the greater the RT value and the smaller the TTP value. In contrast, the larger the proportion of portal vein blood supply, the smaller the RT value, and the greater the TTP value.^[14,16]^ PI and AUC are indicators that reflect tissue microcirculation blood volume and the degree of contrast agent microbubble aggregation, respectively; WIS is an indicator that reflects the richness of tissue microcirculation blood supply and the velocity of contrast agent microbubbles entering and exiting the bloodstream.^[17,18]^ Patients with LNM, late TNM staging, and poor treatment response have a predominantly portal vein blood supply to the lesion. Moreover, the tissue microcirculation blood volume, blood supply richness, contrast agent microbubble aggregation, and inflow and outflow blood flow velocity were higher, and the RT, PI, and AUC values were significantly lower than those of patients without LNM, with earlier TNM staging, and better therapeutic effects.^[12–20]^ However, this study did not find significant differences between TTP and WIS in patients with HCC with different LNM, TNM staging, and treatment response, which differs from the results of Li et al^[21]^ and Luo et al.^[22]^ The reason for the analysis may be related to the small sample size included in this study. From the above results, it can be concluded that changes in RT, PI, and AUC may be related to LNM, TNM staging, and the therapeutic effect on HCC. Further ROC curve analysis revealed that RT, PI, and AUC have certain value in evaluating LNM, TNM staging, and treatment response of HCC. However, there is a problem of low sensitivity or specificity when conducting individual tests. When the 3 indicators were combined, the AUC value, sensitivity, and specificity for LNM, TNM staging evaluation, and treatment response evaluation of HCC were significantly improved compared with single detection. The combination of these 3 factors has a high value in evaluating LNM, TNM staging, and therapeutic treatment response for HCC.^[23]^ However, it should be pointed out that CEUS parameters are easily affected by abdominal gas and respiratory movement in patients. Therefore, during the actual operation, the patient should be instructed to empty the abdomen and maintain stable breathing to avoid the influence of personal factors on the examination results.

CEUS can observe the entire time from liver imaging to the complete disappearance of the contrast agent, and the regression rate of the contrast agent in the HCC lesion is earlier than that of the surrounding normal tissues, so it can carry out a comprehensive scan of the liver under the portal venous phase and delayed phase scans, which is conducive to the detection of small lesions in the liver. Therefore, in clinical practice, CEUS has a high clinical diagnostic value for diagnosing the postoperative recurrence and metastasis of small intrahepatic lesions, and is helpful for the early detection, diagnosis and treatment of postoperative residual and recurrent tumors, thereby improving the treatment effect and patient survival, and is worthy of clinical choice.

*Limitations*: First, this single-center retrospective study included a limited sample size, and patients had a certain selection bias. Second, the CEUS parameters may be affected by human or technical factors. Third, the ultrasound images of patients with different LNM, TNM stages, and curative effects were not analyzed. Finally, the follow-up period was short. In the future, we will continue to conduct higher-quality research to verify this conclusion.

## 5. Conclusion

CEUS parameters, such as RT, PI, and AUC, can be used for LNM, TNM staging, and curative effect evaluation of HCC. The combination of the 3 parameters is feasible and valuable in the evaluation of LNM, pathological staging, and the curative effect of HCC.

## Author contributions

**Conceptualization:** Chenhao Ying, Yefei Yao.

**Formal analysis:** Haijing Song.

**Investigation:** Binjun Yang.

**Writing – original draft:** Chenhao Ying.

**Writing – review & editing:** Chenhao Ying.
